# Water Quality Evaluation and Countermeasures of Pollution in Wan’an Reservoir Using Fuzzy Comprehensive Evaluation Model

**DOI:** 10.3390/toxics13090712

**Published:** 2025-08-23

**Authors:** Gaoqi Duan, Li Peng, Chunrong Wang, Qiongqiong Lu

**Affiliations:** 1 Center for Water and Ecology, State Key Laboratory of Regional Environment and Sustainability, School of Environment, Tsinghua University, Beijing 100084, China; duangaoqi@tsinghua.edu.cn; 2 Tai’an Engineering Construction Guidance Service Center, Tai’an 271002, China; pengli8168@ta.shandong.cn; 3 School of Chemical and Environmental Engineering, China University of Mining and Technology (Beijing), Beijing 100083, China; wcr@cumtb.edu.cn; 4 State Key Laboratory of Soil Pollution Control and Safety, Chinese Academy of Environmental Planning, Beijing 100041, China

**Keywords:** water quality evaluation, fuzzy comprehensive evaluation, pollutant emissions, impact sources, spatial variations

## Abstract

Water quality evaluation is a crucial component of water source management and pollution prevention, essential for achieving regional water safety and sustainable development. The spatial distribution and trends of major water pollutants in Wan’an Reservoir were analyzed. Subsequently, a fuzzy membership model was employed to develop a comprehensive water quality evaluation method. This approach assessed spatial variations in water quality across the upper, middle, and lower reaches of the reservoir, identifying key factors influencing water quality. The results indicate that water quality in Wan’an Reservoir, primarily characterized by total nitrogen, was poor. Notably, 50% of the sampling points in the main stream were identified as highly polluted, with the highest exceedance rate observed in the middle reaches of the tributaries. Sampling points classified as Class I were predominantly located in the upper reaches, where water quality benefitted from clean incoming water and minimal disturbance. In contrast, the lower reaches experienced more severe pollution due to the cumulative effects of domestic sewage, industrial wastewater, and agricultural runoff. These findings are crucial for developing effective water environmental protection strategies and promoting the sustainable utilization and protection of water resources.

## 1. Introduction

Rapid industrialization and urbanization have placed unprecedented pressure on water resources [1,2,3]. Water pollution, particularly in freshwater resources such as rivers and lakes, has become a global environmental issue, severely threatening human health, ecological balance, and sustainable development [4,5,6]. Therefore, formulating effective water environment protection strategies and promoting the rational use and preservation of water resources are crucial. Water quality assessment is a key prerequisite for developing scientifically sound strategies.

Water quality assessment, a crucial component of water environment management, aims to evaluate the pollution status, trends, and potential risks of water bodies through monitoring and analysis of various water quality indicators [7,8]. Commonly used water quality evaluation methods include the single-factor evaluation method, gray correlation method, Bayesian evaluation method, artificial neural network method, and fuzzy comprehensive evaluation method [9,10,11,12,13]. The single-factor evaluation method intuitively identifies the main pollution factors at monitoring sites and selects the worst water quality index to determine the water quality category, but it does not provide an objective assessment of the overall water quality [14,15]. The gray correlation method is highly flexible and applicable to systems with limited data, but it requires advanced expertise and involves significant uncertainty [16,17]. The Bayesian evaluation method is simple to calculate and operate, but it requires careful sample selection, and the results can be influenced by sample position and quantity [18,19]. In comparison, the fuzzy comprehensive evaluation method accounts for both the degree of each water quality value and the weight of pollution factors, addressing uncertainties and providing a more concise and efficient reflection of the overall water quality in the basin [20,21]. Wang et al. proposed and established a comprehensive pollution evaluation system based on an entropy weight–fuzzy evaluation model, integrating overlying water and sediment to accurately assess the pollution status of urban rivers [22]. Xu et al. applied the fuzzy comprehensive evaluation method to assess the water quality of Nansi Lake Basin in China, providing valuable insights for its water quality evaluation [23]. Zhang et al. developed a groundwater quality assessment model by integrating the entropy weight method with an improved fuzzy comprehensive evaluation approach based on water quality data from 14 boreholes in Cengong County, Guizhou Province. Coupled with GIS technology, they assessed the spatial distribution of aquifer water quality in the region. The results demonstrated that the proposed method is both feasible and effective for evaluating groundwater quality [24]. However, the fuzzy comprehensive evaluation method is currently mainly applied to specific rivers, lakes, and groundwater systems, with limited application to reservoir basins. In recent years, with the continued industrialization of China, Jiangxi Province, known for its relatively better water environment, has been facing worsening water pollution. Wan’an Reservoir, situated in the middle and upper reaches of the Ganjiang River Basin, is an artificial lake formed by damming the Ganjiang River and houses the largest hydropower station in Jiangxi Province [25]. While it plays a significant role in promoting economic and social development, it may also lead to various environmental issues [26]. As the sole source of drinking water for Wan’an County and some surrounding townships, the water quality and ecological safety of this water conservancy project directly impact the drinking water safety and ecological health of the surrounding areas [27,28]. To avoid repeating the mistakes of “pollution before treatment,” it is crucial to guide the coordinated and sustainable development of both economic growth and ecological protection. This requires actively promoting the national and Jiangxi provincial water pollution prevention plans and evaluating the water quality of Wan’an Reservoir. Based on the above, our major aims were (i) to analyze the spatial distribution and trends of the main water pollutants of Wan’an Reservoir; (ii) to develop a comprehensive water quality evaluation method and measure spatial changes in water quality across the upper, middle, and lower reaches of Wan’an Reservoir; and (iii) to calculate pollutant emissions around Wan’an Reservoir and provide a scientific basis for effective water quality management and protection measures.

## 2. Materials and Methods

### 2.1. Study Area

**Figure 1:**
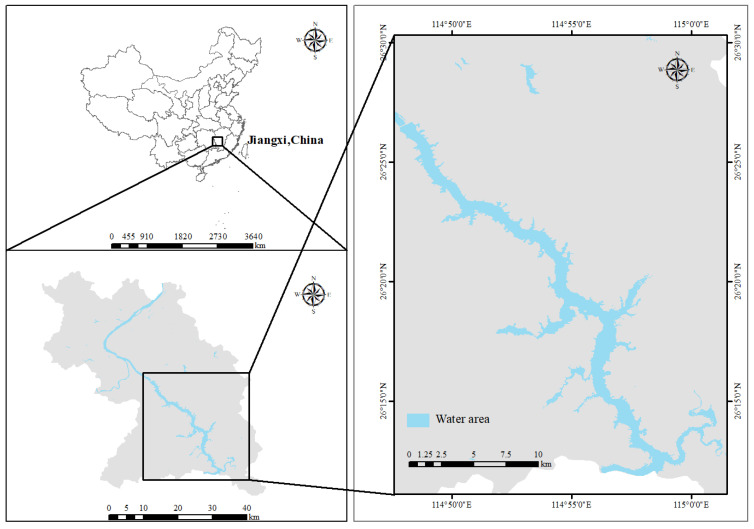
Location map of Wan’an Reservoir.

Wan’an Reservoir, situated in the middle and upper reaches of the Ganjiang River Basin, is an artificial lake formed by damming the Ganjiang River and houses the largest hydropower station in Jiangxi Province. Since its construction and impoundment, the reservoir extends from Furong Town, Wan’an County, in the north to Bajingtai, Ganzhou City, in the south (Figure 1). The catchment area of Wan’an Reservoir is 36,900 km^2^, accounting for 44.56% of the Ganjiang River Basin [29]. The reservoir has a water surface area of 107.5 km^2^, a total capacity of 2.214 billion m^3^, an average water depth of 96 m, and a designed flood level of 100 m.

**Figure 2:**
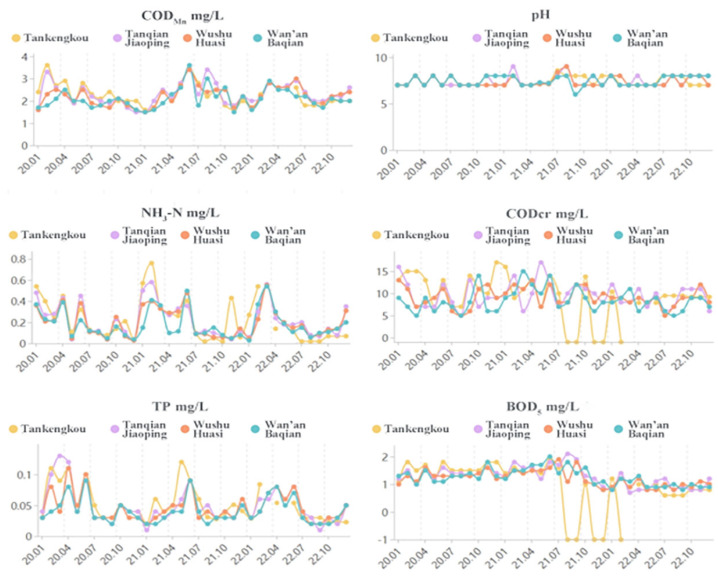
Trends in major pollutants at key water quality monitoring sections in Wan’an County from 2020 to 2022.

**Figure 3:**
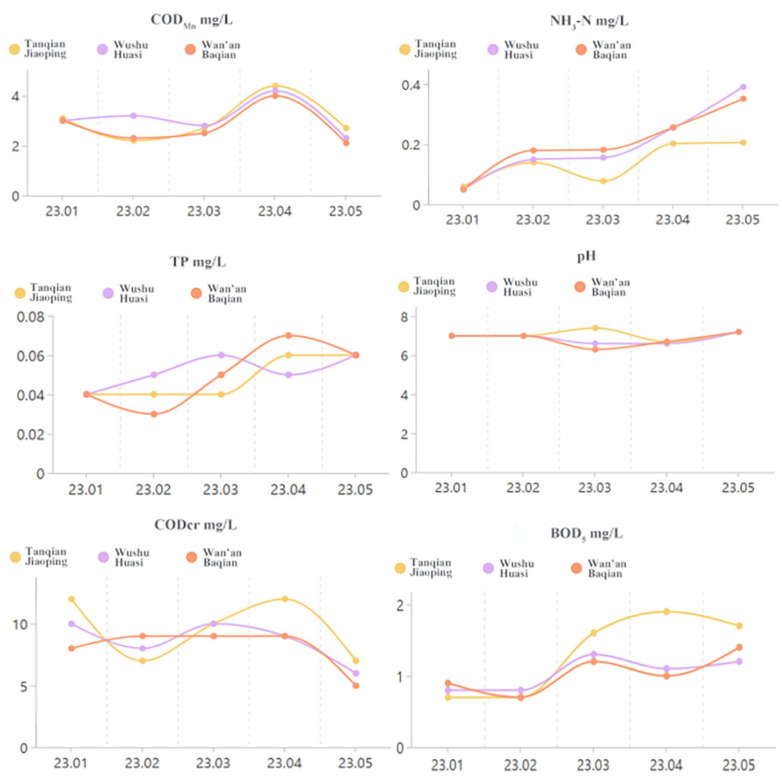
Trends in major pollutants at key water quality monitoring sections in Wan’an County in 2023.

The monitoring sections of the Wan’an River include Tan Kengkou, Tanqian Jiaoping, Wushu Huasi, and Wan’an Baqian, with monitoring conducted once a month by the Ji’an City Environmental Protection Monitoring Station, as shown in Figure 2 and Figure 3. Analysis of water quality monitoring data from 2020 to 2023 shows that the monitoring sections at Tan Kengkou, Tanqian Jiaoping, Wushu Huasi, and Wan’an Baqian exceeded total phosphorus (TP) standards in February to June of 2020, 2021, and 2023. However, the total phosphorus levels exceeded the standards in February to June of the first half of each year, resulting in Class IV and Class V water quality. All water quality indicators at each monitoring section met the requirements of Class III water quality standards specified in the “Surface Water Environmental Quality Standards” (GB 3838-2002) [30]. Specifically, from 2020 to 2022, the annual average values of chemical oxygen demand (CODcr), ammonia nitrogen (NH_3_-N), and biochemical oxygen demand (BOD_5_) at all monitoring sections in Wan’an County met the Class III water quality standards for surface water; however, the total phosphorus indicator was unstable, with the annual average value only meeting the Class IV water quality standards. In 2023, the BOD_5_, TP, and NH_3_-N values in Wan’an Reservoir showed a fluctuating upward trend, but the annual changes in the average concentrations of each indicator were not significant. Further analysis shows that the water quality indicators in this area are relatively stable from September to November each year, and the results are more representative. Therefore, the sampling period for this study is from September to November 2023.

### 2.2. Data Sources

**Figure 4:**
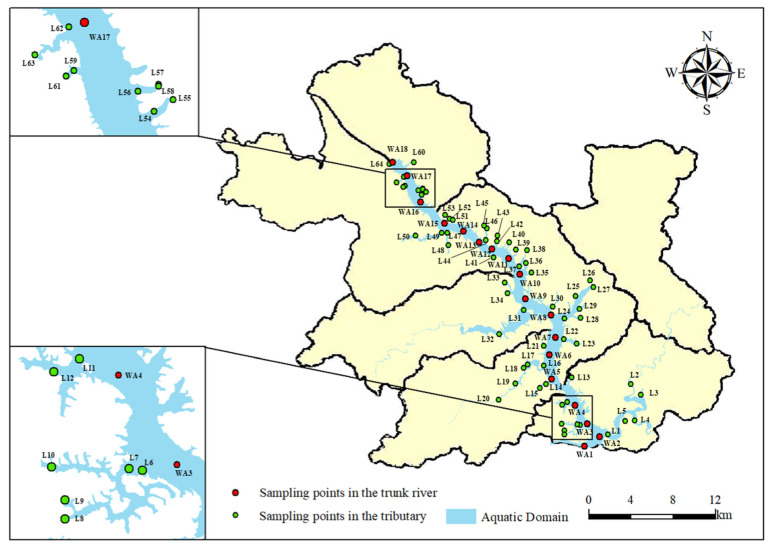
Map of sampling points for water quality investigation in Wan’an Reservoir Basin.

A sampling investigation on the water quality of Wan’an Reservoir and its rivers was conducted from September to November 2023. The investigation included 18 reservoir areas, 64 tributary sites, and control points. The specific monitoring points are depicted in Figure 4. The sampling sites are located in the Wan’an Reservoir Basin, Wan’an County. Rural houses and large-scale aquaculture farms are scattered along both sides of the main stream. Near the tributary sampling points, rural houses, aquaculture farms, livestock and poultry farms, and agricultural planting areas are also present. The water quality index was analyzed and determined using the basic item analysis method specified in the “Environmental Quality Standard of Surface Water” (GB3838-2002). Monitoring sections were set at 5 m intervals along the main stream of Wan’an Reservoir, with sampling points located 0.5 m below the water surface. Water was collected from each sampling point using a 2 L plexiglass intake device (Zealquest Scientific Technology Co., Ltd., Shanghai, China). The field index was then determined, and the water samples were transferred to sample bottles and stored in portable collection boxes. A YSI multi-parameter water quality detector (Vesai Instrument Trading Co., Ltd., Shanghai, China) was used to record real-time parameters, including water sample temperature, pH, dissolved oxygen, conductivity, redox potential, total suspended solids, and other variables. After on-site sampling, the water samples were transported to the laboratory and stored in a 4 °C incubator. The determination and analysis of dissolved oxygen (DO), permanganate index, ammonia nitrogen, total phosphorus, total nitrogen, and heavy metals were then conducted. Based on the available monitoring data, DO, permanganate index (COD_Mn_), TN, NH_3_-N, and TP were selected to evaluate the water quality of the main stream and tributaries of Wan’an Reservoir.

### 2.3. Fuzzy Comprehensive Evaluation Model

(1) Determining index weight by using entropy weight method

Due to the differences in dimensions and orders of magnitude among the indicators, the data are first standardized and then converted into dimensionless values. By mapping the original data to the [0, 1] interval, the formula eliminates the dimensional influence between indices, making each index comparable in subsequent calculations. The min-max standardization formula is used as follows:

For a positive indicator:

(1) $$x_{ij}^{*}=\frac{x_{ij}-x_{min}}{x_{max}-x_{min}}$$

For a negative indicator:

(2) $$x_{ij}^{*}=\frac{x_{max}-x_{ij}}{x_{max}-x_{min}}$$

where $x_{ij}$ is the original data, $x_{ij}^{*}$ is the standardized data, and $x_{jmin}$ and $x_{jmax}$ are the minimum and maximum values of the *j*-th indicator, respectively.

The information entropy of each index is then calculated based on the standardized data. Information entropy reflects the degree of disorder or uncertainty in the index. A smaller entropy value indicates greater information provided by the index, resulting in a higher weight. The formula, derived from information theory’s measure of uncertainty, calculates the information entropy through logarithmic operations and weighted summation of each sample’s proportion to the index. The formula for calculating information entropy is as follows:

(3) $$E_{j}=-k\sum_{i=1}^{n}\;p_{ij}\ln\left(p_{ij}\right)$$

where n is the number of samples. $p_{ij}=\frac{x_{ij}^{*}}{\sum_{i=1}^{n}\;x_{ij}^{*}}$, $p_{ij}$ represents the proportion of the i-th sample under the j-th index, and $k=\frac{1}{ln(n)}$.

The weight of each index is calculated via information entropy, and the calculation formula is as follows:

(4) $$w_{j}=\frac{1-E_{j}}{\sum_{j=1}^{m}\;\left(1-E_{j}\right)}$$

where m is the number of indicators.

The specific weight of each index is provided in Table 1.

(2) Construction of fuzzy membership model

To reflect the differences in evaluation results, the water quality evaluation index for each monitoring criterion is divided into five grades: I, II, III, IV, and V. As a drinking water source, Wan’an Reservoir is required to comply with relevant regulatory standards. “Sanitary Standard for Drinking Water” (GB 5749-2022) [31] stipulates that when surface water serves as a drinking water source, it must satisfy all criteria specified in “Environmental Quality Standards for Surface Water” (GB 3838-2002). To ensure comparability and practical guidance, the threshold values for water quality evaluation indices corresponding to DO, TN, NH_3_-N, TP, and COD_Mn_ are determined, referring to the “Environmental Quality Standards for Surface Water” (GB 3838-2002) (Table 1).

Fuzzy membership functions are constructed for each evaluation index and corresponding evaluation grade. A reduced semi-trapezoidal distribution function is used to describe the degree of membership to different water quality grades. In this study, both semi-trapezoidal and triangular fuzzy membership functions are primarily used:

Triangular fuzzy membership function:

(5) $$r_{ij}=\left\{\begin{array}{ll} 0, & x\leq a\\ \frac{x-a}{m-a}, & a\leq x\leq m\\ \frac{b-x}{b-m}, & m\leq x\leq b\\ 0, & x>b \end{array}\right.$$

Semi-trapezoidal fuzzy membership function:

For a positive indicator:

(6) $$r_{ij}=\left\{\begin{array}{ll} 1, & x\leq a\\ \frac{x-a}{b-a}, & a\leq x\leq b\\ 0, & x>b \end{array}\right.$$

For a negative indicator:

(7) $$r_{ij}=\left\{\begin{array}{ll} 1, & x\leq a\\ \frac{b-x}{b-a}, & a\leq x\leq b\\ 0, & x>b \end{array}\right.$$

Using the constructed fuzzy membership functions, the membership degree of each sample in relation to different water quality grades under each index is calculated, resulting in the fuzzy membership matrix R (Table S1). The elements $r_{ij}$ of matrix R represent the membership degree of the i-th sample to the k-th water quality grade under the j-th index.

(8) $$R=\left[\begin{array}{ccc} r_{11} & \ldots & r_{1j}\\ \vdots & \ddots & \vdots \\ r_{i1} & \ldots & ij \end{array}\right]$$

(3) Comprehensive evaluation

The index weight vector W, determined using the entropy weight method, and the fuzzy membership matrix R are weighted to obtain the comprehensive evaluation vector B, as follows:

(9) B=W×R

The elements bk of the comprehensive evaluation vector B represent the overall membership degree of the samples to the k-th water quality grade. The water quality grade of each sample is determined based on the principle of maximum membership degree.

## 3. Results

### 3.1. Spatial Variation Characteristics of Water Quality Index in the Main Stream of Wan’an Reservoir

The descriptive statistics of water quality parameters across 83 sampling sites are presented in Table 2. Dissolved oxygen (DO) concentrations exhibited a broad range from 4.89 to 18.36 mg/L, with a median value of 9.25 mg/L exceeding the Class III surface water standard (≥5 mg/L). The substantial standard deviation (2.567 mg/L) indicates significant spatial heterogeneity, likely attributable to localized aeration processes or pollution sources. Total nitrogen (TN) demonstrated extreme variation, spanning from below the detection limit to 18.94 mg/L—approximately 8.5 times higher than the Class V threshold. While the median TN concentration (1.05 mg/L) meet Class IV standards, the pronounced standard deviation (3.714 mg/L) confirms the presence of high-intensity pollution hotspots.

Ammonia nitrogen (NH_3_-N) levels ranged from 0.020 to 2.74 mg/L, with a median of 0.18 mg/L, conforming to Class II criteria. The relatively low standard deviation (0.271 mg/L) suggests generally stable spatial distribution, though maximum values indicate severe ammonium contamination in discrete locations. Total phosphorus (TP) concentrations varied between 0 and 0.300 mg/L, with a median (0.022 mg/L) satisfying Class II requirements. The minimal standard deviation (0.031 mg/L) implies that diffuse phosphorus sources dominate the study area. The permanganate index (COD_Mn_) displayed moderate fluctuation (0.000–4.84 mg/L), with a median value of 1.972 mg/L, approaching the Class III upper limit, and a standard deviation of 1.236 mg/L, reflecting spatially heterogeneous organic pollution. Notably, the extreme variability in TN (Coefficient of Variation = 3.54) and COD_Mn_ (CV = 0.63) substantiates the necessity of entropy-weighted modeling to address parameter sensitivity.

**Figure 5:**
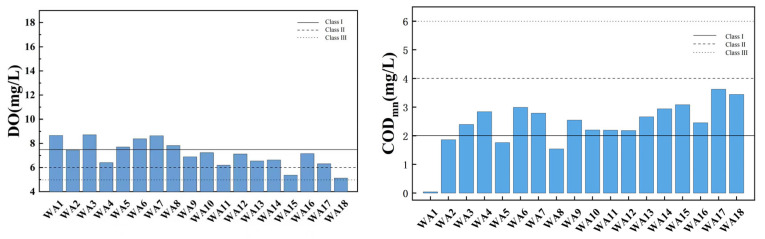

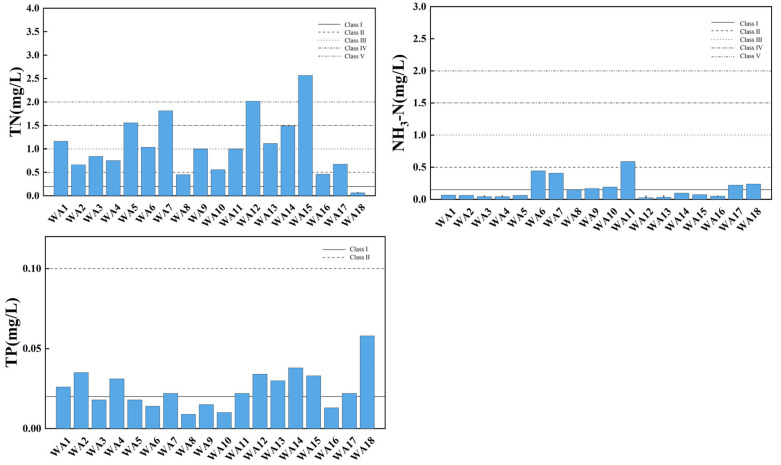
Spatial variation characteristics of water quality index in main stream of Wan’an Reservoir.

Figure 5 illustrates the spatial variation of the water quality index in the main stream of Wan’an Reservoir. DO in water is closely related to the presence of organic matter, which consumes oxygen. Higher dissolved oxygen content typically indicates better water quality [32,33]. A low dissolved oxygen content may indicate the presence of pollutants or excessive organic matter. Dissolved oxygen levels are mostly above the Class II water standard, with sampling points WA1, WA3, WA5, WA6, WA7, and WA8 classified as Class I water quality, and WA15 and WA18 classified as Class III. DO decreases slightly downstream, fluctuating between 5.13 mg/L and 8.72 mg/L, with an average of 7.14 mg/L. The reasons for this trend may be related to the flow of water also affecting oxygen dissolution. Flowing water absorbs oxygen from the atmosphere more easily, so dissolved oxygen levels may be higher upstream. COD_Mn_ is commonly used to reflect pollution levels of organic and inorganic oxidizable substances in water [34,35]. All sampling points have a permanganate index within Class II, indicating relatively good water quality. Four sampling points meet Class I standards, suggesting lower permanganate content and superior water quality. The permanganate index measures organic and oxidative pollution in water, with lower values typically associated with better water quality. The average permanganate index is 2.42 mg/L, indicating overall good water quality. The permanganate index increases slightly downstream, likely due to organic matter, suspended solids, and other factors [36,37]. TN, including all forms of organic and inorganic nitrogen (e.g., organic nitrogen, ammonia, protein), is an important water quality indicator. The total nitrogen index shows significant spatial variation, with 50% of main stream sampling points being key polluted areas, and WA12 and WA15 classified as Class V. NH_3_-N refers to nitrogen in the form of free ammonia and ammonium ions in water. Ammonia nitrogen levels meet Class II standards at all points except WA11, with 55.56% of points meeting Class I standards. The TP index at all sampling points is stable, fluctuating between 0.009 and 0.058 mg/L, with an average of 0.025 mg/L, all meeting Class II standards.

### 3.2. Spatial Variation Characteristics of the Water Quality Index at the Inlet and Control Point of Tributaries of Wan’an Reservoir

**Figure 6:**
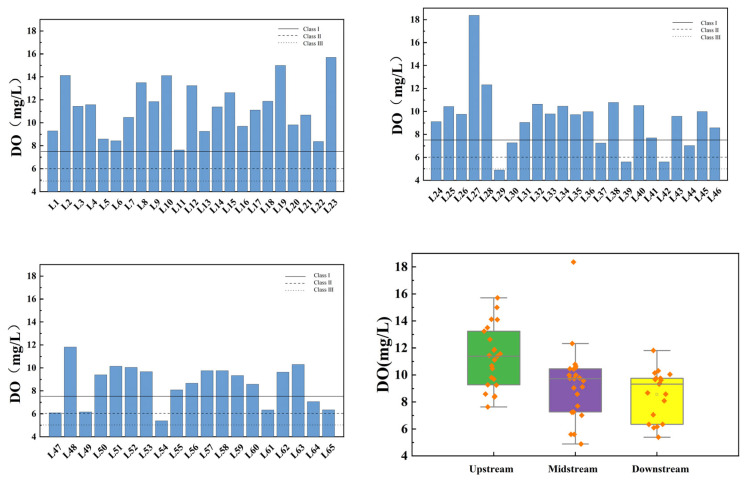
Spatial variation characteristics of dissolved oxygen at inflow point and control point of tributaries of Wan’an Reservoir.

Figure 6 illustrates the spatial variation of DO at the inflow and control points of the tributaries of Wan’an Reservoir. The average DO values in the upstream, midstream, and downstream sections of the tributaries are 11.29 mg/L, 9.32 mg/L, and 8.55 mg/L, respectively. Both upstream and downstream sections meet Class III standards and generally exceed Class II standards. The midstream section meets Class II water standards, except for L29 Jiangbei Reservoir, with an overall compliance rate exceeding 98%.

**Figure 7:**
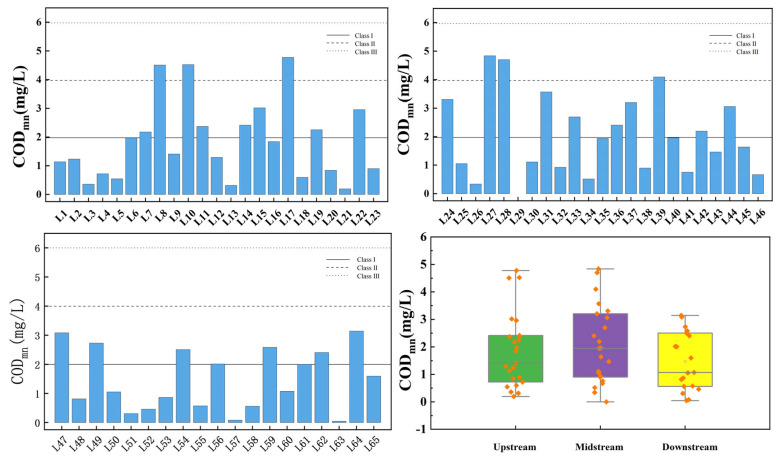
Spatial variation characteristics of permanganate index at inflow point and control point of tributaries of Wan’an Reservoir.

Figure 7 presents the spatial variation of the permanganate index at the inflow and control points of the tributaries of Wan’an Reservoir. The average permanganate index values for the upstream, midstream, and downstream sections are 1.842 mg/L, 2.059 mg/L, and 1.469 mg/L, respectively. The permanganate index at all sampling points complies with Class III water standards. The compliance rate for Class II water standards in the upstream and midstream sections is 86.96%, while the compliance rate for Class I water standards is 56.52%. All downstream sampling points meet Class II water standards, with a compliance rate of 57.89% for Class I standards. The compliance rate for the overall Class II water standard is 90.77%, while that for Class I water standard is 56.92%. The box plot indicates that the permanganate index in the downstream section is at an excellent level.

**Figure 8:**
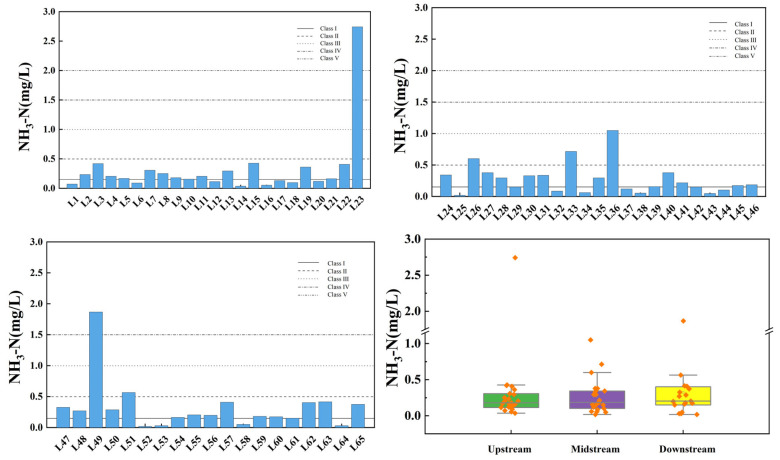
Spatial variation characteristics of ammonia nitrogen at inflow point and control point of tributaries of Wan’an Reservoir.

Figure 8 illustrates the spatial variation of ammonia nitrogen at the inflow and control points of the tributaries of Wan’an Reservoir. The average ammonia nitrogen concentrations in the tributaries of Wan’an Reservoir are 0.314 mg/L in the upstream, 0.271 mg/L in the midstream, and 0.321 mg/L in the downstream sections, respectively. The exceedance rate in the upper and middle reaches is 4.35%, while in the downstream section, it is 5.26%. In each flow section, one sampling point fails to meet the Class III water standard: the east side of L23 (control point), the area behind L36 tower, and the right corner bay of L49. These monitoring sites are primarily distributed in Wushu Township, where aquaculture is relatively well developed. Previous studies have indicated that animal feedlots and crop cultivation activities are the main sources of NH_3_-N [38].

**Figure 9:**
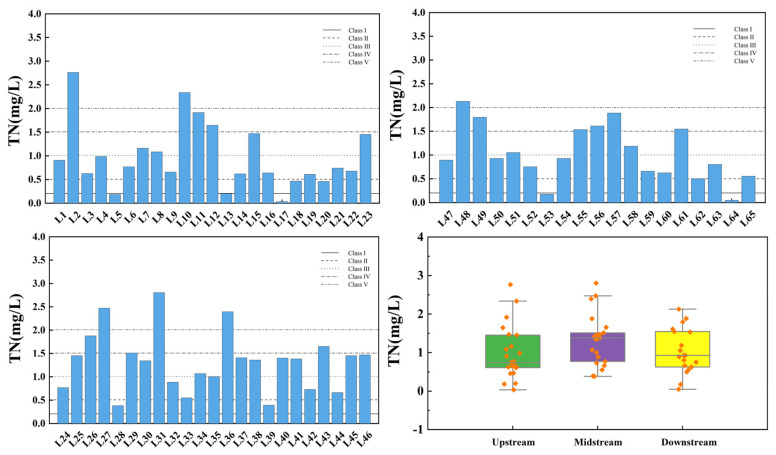
Spatial variation characteristics of total nitrogen at inflow point and control point of tributaries of Wan’an Reservoir.

Figure 9 shows the spatial variation of total nitrogen at the inflow and control points of the tributaries of Wan’an Reservoir. The TN index of tributary sampling points exhibits significant spatial variability, with the exceedance rates in the upstream, midstream, and downstream sections being 34.78%, 65.22%, and 42.11%, respectively. All sections of the reservoir’s tributary basin are severely polluted. Overall, 23.08% of the sampling points exceed the Class IV standard, and 9.23% exceed the Class V standard.

**Figure 10:**
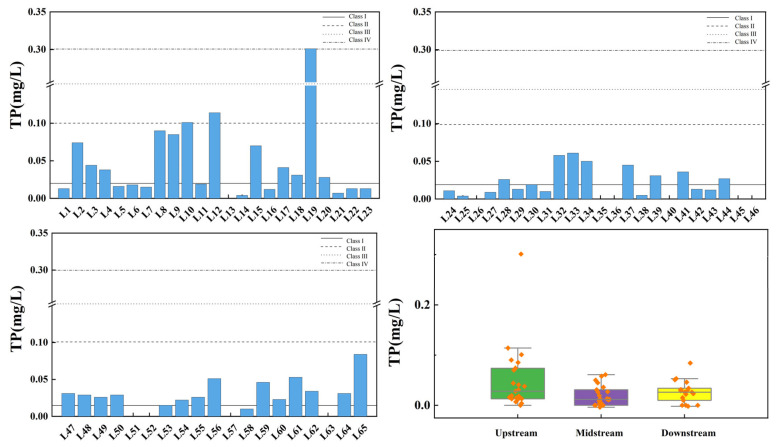
Spatial variation characteristics of total phosphorus at inflow point and control point of tributaries of Wan’an Reservoir.

Figure 10 illustrates the spatial variation of TP at the inflow and control points of the Wan’an Reservoir tributaries. TP in tributaries comprises both inorganic and organic phosphorus. P is a key element for plant growth, and appropriate levels promote the growth and reproduction of aquatic organisms. However, excessive P concentration disrupts the aquatic ecosystem’s balance [39,40]. In the Wan’an Reservoir tributary, the average TP concentration in the upstream section is 0.050 mg/L, while at control point 3 (L19 pool), it reaches 0.301 mg/L, exceeding the Class III water standard. The exceedance rate in the upstream section is 4.35%. The average TP concentration in the middle reaches is 0.018 mg/L, meeting the Class II standard, with 65.22% of points meeting the Class I standard. The average TP concentration in the downstream section is 0.027 mg/L, meeting the Class II standard, with 31.58% of points meeting the Class I standard.

### 3.3. Spatial Variation Analysis Using Fuzzy Comprehensive Evaluation Index of Water Quality

(1) Fuzzy comprehensive evaluation of the main stream of Wan’an Reservoir

**Figure 11:**
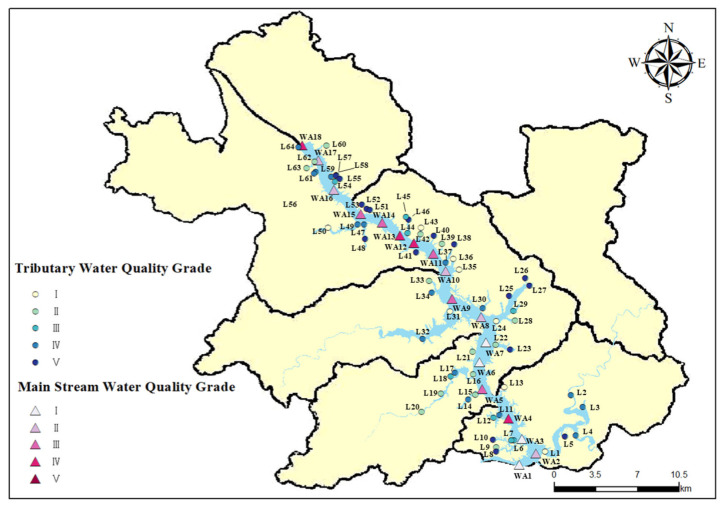
Spatial distribution of water quality of Wan’an Reservoir.

The water quality monitoring results of the main and tributary streams of Wan’an Reservoir exhibit notable variations and complexity. The specific results of the water quality assessment are presented in Figure 11. In the main stream area, 18 sampling points (WA1–WA18) were analyzed. Of these, four sampling points (WA1, WA3, WA6, and WA7) were classified as Grade I, representing approximately 22.2% of the total, indicating “good” water quality. These areas are characterized by high DO levels (e.g., WA1: 8.67 mg/L) and low COD_Mn_ (e.g., WA1: 0.04 mg/L), suggesting a strong self-purification capacity and minimal pollution. This may be attributed to better water quality in upstream areas, reduced human interference, and a relatively stable ecological environment. Five sampling points (WA2, WA8, WA10, WA16, and WA17) were classified as Grade II, representing 22.2% of the total, with “good” water quality. Five sampling points (WA5, WA9, WA11, WA14, and WA15) were rated as Grade III, accounting for 22.2% of the total, reflecting “average” water quality. Four sampling points (WA4, WA12, WA13, and WA18) were classified as Grade IV, accounting for 11.1%, indicating “poor” water quality. Notably, no sampling points in the main stream were classified as Grade V, which denotes “very poor” water quality. The water quality at sampling point WA18 is classified as Grade IV, with TN reaching 0.064 mg/L, TP at 0.058 mg/L, and COD_Mn_ at 3.443 mg/L. These values indicate significant pollution, likely caused by the cumulative effects of domestic sewage, industrial wastewater inflow, and agricultural runoff in upstream densely populated areas [41]. This pollution exceeds the natural self-purification capacity of the water body, leading to significant deterioration of water quality.

Spatially, the water quality in the upper reaches of Wan’an Reservoir is notably better than in the lower reaches. Sampling points with higher water quality (Grade I and II) are predominantly located in the upstream section of the main stream. The frequency of low scores (Grades IV and V) increases downstream, indicating a clear trend of water quality deterioration along the river. This trend is linked to the natural flow characteristics of rivers and the distribution of pollution sources. The water quality of the downstream sampling point WA18 was classified as Grade IV, possibly due to cumulative pollution. A small number of rural houses are located near WA13 and WA14, with a high concentration of fishing boats around WA13. These factors likely contribute to the poor water quality observed at these sampling points. The improvement in water quality at sampling points downstream of WA8 may be attributed to the confluence of two tributaries, L24 and L31.

(2) Fuzzy comprehensive evaluation of tributaries of Wan’an Reservoir

Among the 60 sampling points (L1–L60) in tributaries, the distribution is as follows: 9 Class I points (13.8%), 15 Class II points (23.1%), 8 Class III points (12.3%), 16 Class IV points (24.6%), and 17 Class V points (26.2%). Overall, the distribution of sampling points across grades in tributaries is more dispersed compared to the main stream. The total number of Grade I and II sampling points is similar in both tributaries and the main stream, but tributaries show a more balanced distribution of Grade III to V points. Grade I monitoring points, such as L1, exhibit higher DO, with L1 reaching 9.28 mg/L, and lower concentrations of major pollutants. These sampling points are typically located near the source of tributaries or in regions less influenced by human activities, where the surrounding ecological conditions support high water quality. In contrast, monitoring points such as L8, L10, and L27 are classified as Grade V due to poorer water quality. For instance, L27 has a TN concentration of 12.34 mg/L and a COD_Mn_ level of 4.84 mg/L. These sites are typically located in the lower reaches of tributaries or pass through densely populated areas, agricultural zones, and intensive aquaculture regions. They are heavily impacted by domestic sewage, agricultural runoff (including chemical fertilizers and pesticides), and wastewater from fishery aquaculture, leading to significant water quality deterioration.

A correlation exists between the water quality scores of certain tributaries at sampling points near the confluence with the main streams and the water quality scores at corresponding points on the main streams. In areas where water quality is poor, nearby tributaries often have lower water quality scores. This could result from the backflow of polluted water from the main stream into the tributaries or the diffusion of pollutants from the main stream, leading to a synergistic pollution effect in these local regions [42]. Specifically, the tributaries corresponding to L47 and L48, as well as the tributary corresponding to L39, exhibit poor water quality scores. This pattern is consistent with the water quality of the main stream at these corresponding locations.

(3) Analysis of Fuzzy Comprehensive Evaluation Results

**Figure 12:**
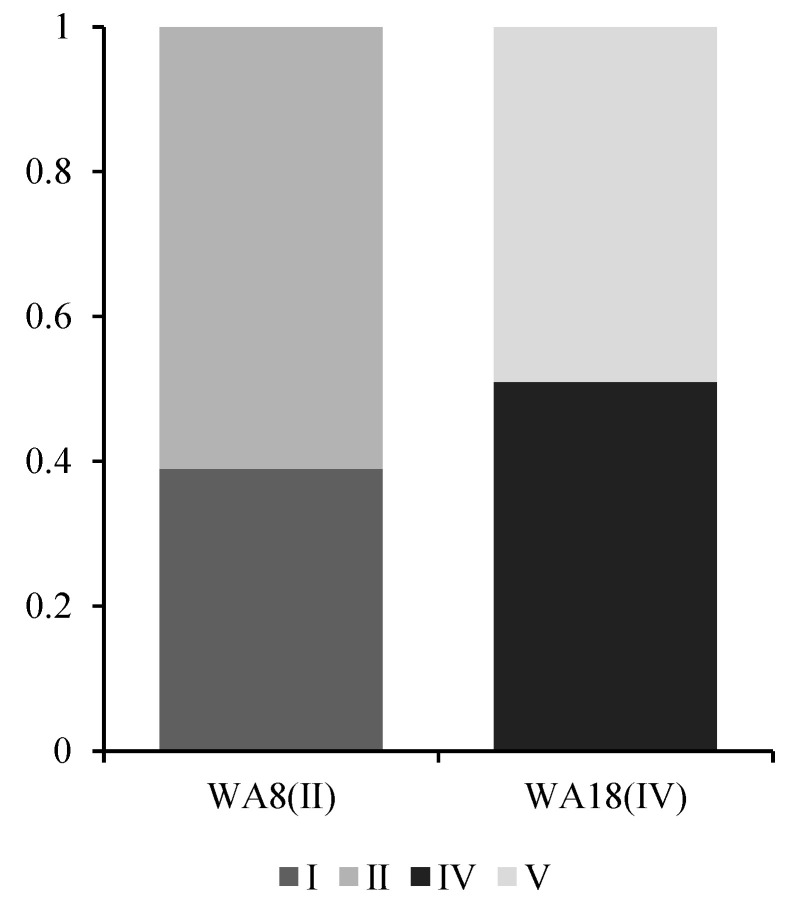
Normalized membership diagram for selected ambiguous sampling points.

The membership matrix serves as the fundamental basis for fuzzy comprehensive evaluation, yet its inherent uncertainty warrants attention. When the difference between the second highest and highest membership degrees falls below 0.2, we consider the water quality assessment at that point to exhibit ambiguity. Figure 12 illustrates two representative sampling points with such ambiguous evaluations, where their membership degrees were normalized. Although the maximum member principle was applied in previous assessments to determine grades, situations similar to those described in the figure (i.e., situations that generate assessment uncertainty) must not be ignored.

**Figure 13:**
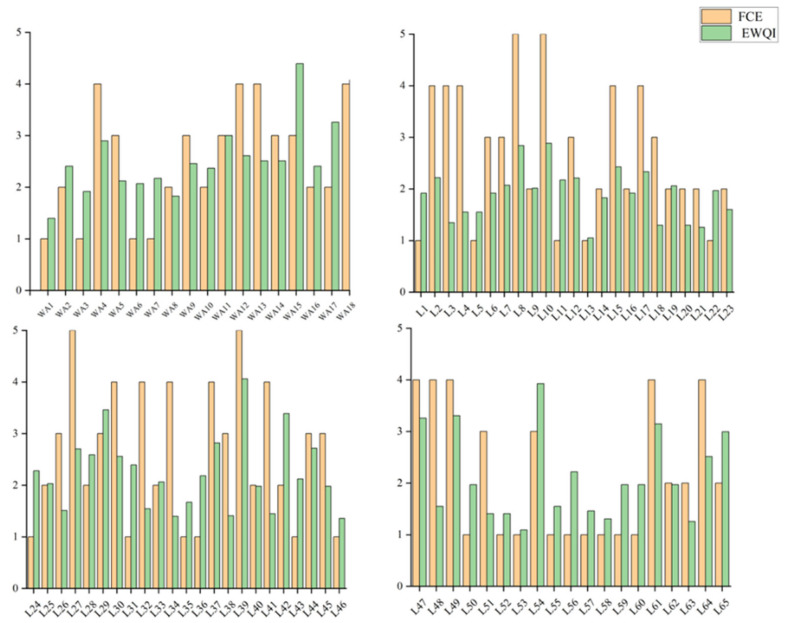
Comparison of water quality assessment between entropy weight–fuzzy comprehensive evaluation and EWQI.

After evaluating the study area using the entropy-weighted water quality index (EWQI) and comparing it with the entropy weight–fuzzy comprehensive evaluation, the results are shown in Figure 13. It can be observed that the entropy weight–fuzzy comprehensive evaluation method yields more pessimistic water quality assessments, though the overall results align with those from the EWQI. Taking sampling point L21 as an example, the entropy weight–fuzzy comprehensive evaluation classified the water quality as Class V, while the EWQI indicated Class I. For the L8 water sample, despite its high dissolved oxygen (DO) content, the fuzzy comprehensive evaluation revealed underlying issues masked by this favorable indicator—with TN, NH_3_-N, TP, and COD_Mn_ concentrations reaching 4.54 mg/L, 0.25 mg/L, 0.09 mg/L, and 4.51 mg/L, respectively.

### 3.4. Analysis of Water Quality Impact Sources of Wan’an Reservoir

**Figure 14:**
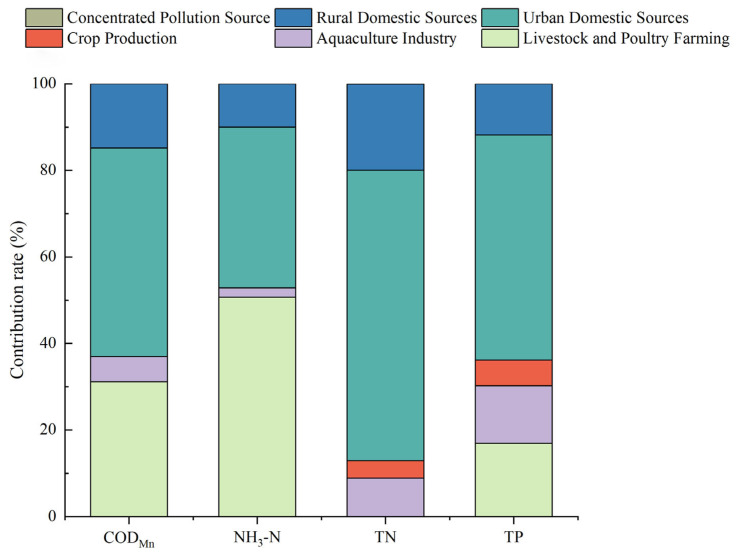
Contribution ratios for COD_Mn_, NH_3_-N, TN, and TP in Wan’an Reservoir.

The pollutant emissions around Wan’an Reservoir were calculated based on the accounting methods and related parameters outlined in several key documents, including the “Technical Guide for Compilation of Total Amount Control Plan of Major Pollutants in the Twelfth Five-Year Plan”, the “Pollutant Emission Standards for Livestock and Poultry Breeding Industry”, the “Handbook of Accounting Coefficients for Agricultural Sources”, the “Handbook of Accounting Coefficients for Domestic Sources”, and the “Handbook of Emission Coefficients for Centralized Pollution Control Facilities”. The results are presented in Table 3. Table 3 shows that the annual COD_Mn_ discharge around Wan’an Reservoir totals 3681.8128 t/a, with 1359.0193 t originating from agricultural sources and 2321.7905 t from domestic sources. The annual ammonia nitrogen emissions are 457.7612 t/a, with 241.6648 t from agricultural sources and 215.9948 t from domestic sources. The TN emissions amount to 348.5136 t/a, with 44.9135 t from agricultural sources and 303.3163 t from domestic sources. The TP emissions are 42.8556 t/a, with 15.4802 t from agricultural sources and 27.3592 t from domestic sources. The contributions of livestock and poultry farming and urban domestic sources to the pollution loads of COD_Mn_ and NH_3_-N are relatively high, while the pollution loads of TN and TP are mainly attributed to urban and rural domestic sources [43,44] (Figure 14).

The water quality of the main stream and tributaries of Wan’an Reservoir shows significant spatial heterogeneity and grade differentiation. Overall, 15.7% of the sampling points fall under Grade I, 24.1% under Grade II, 15.7% under Grade III, 24.1% under Grade IV, and 20.5% under Grade V. Of the 18 sampling points in the main stream, 22.2% were Grade I, mostly concentrated in the upstream, where water quality benefits from good incoming water and minimal interference. Grade II accounted for 27.8%, Grade III for 27.8%, and Grade IV for 22.2%. Pollution downstream is severe. For instance, sampling points such as WA18 are influenced by domestic sewage, industrial wastewater, and agricultural non-point source pollution, with many pollutants exceeding acceptable concentrations. Of the 60 sampling points in the tributaries, 13.8% were Grade I, most of which are located at the source or in areas less affected by human activities. Grade II accounted for 23.1%, Grade III for 12.3%, Grade IV for 24.6%, and Grade V for 26.2%. The distribution of grades is more varied in the tributaries than in the main stream. The downstream areas and tributaries, which pass through densely populated, agriculturally developed, and aquaculture-intensive zones, are heavily polluted. Points such as L8, L10, and L27 are impacted by domestic sewage, agricultural runoff, and fishery wastewater.

From a spatial distribution perspective, the main stream shows a clear trend of water quality deterioration downstream, closely linked to the river’s natural flow characteristics and the distribution of pollution sources [45,46]. Water quality is higher upstream and deteriorates downstream. Additionally, the confluence of some tributaries influences local water quality. Tributaries are highly influenced by local factors, with good water quality at their sources but significant pollution downstream. Furthermore, tributaries are impacted by water diversion, reverse irrigation, or the diffusion of polluted water from the main stream at their confluence [47]. This creates a synergistic local pollution effect, leading to poor water quality scores in tributaries adjacent to degraded areas of the main stream.

Overall, the water quality of the main stream of Wan’an Reservoir varies. The upstream shows better quality while the downstream is significantly polluted. Some sampling points are influenced by human activities. The water quality grades at 60 sampling points in the tributaries vary. The source is generally good, but pollution is prominent in downstream and specific areas, especially near the confluence with the main stream. To enhance the water quality of Wan’an Reservoir, a multi-pronged comprehensive improvement strategy should be implemented. For pollution source control, efforts should focus on improving sewage and pollutant treatment systems in densely populated, industrial, and agricultural areas along the lower reaches of the main stream and tributaries. Discharge of pollutants should be strictly controlled to reduce river contamination. Ecological restoration should prioritize vegetation restoration and constructed wetlands around reservoirs and tributaries. This will enhance ecosystem functions, improve water quality through natural purification, and maintain ecological balance. The monitoring management system should focus on optimizing the network layout, expanding indicator coverage, improving technical standards, and strengthening watershed management and assessment systems. This will ensure accurate water quality monitoring and effective governance.

### 3.5. Limitations

(1) Limitations of Fuzzy Comprehensive Evaluation. The weights in the entropy weight method are mainly based on data dispersion degree. If monitoring data contain unnoticed outliers, this may lead to weighting biases. Additionally, the model assumes that all indicators are independent, without considering the interactions between pollutants. However, in reality, TN and TP may synergistically influence eutrophication, potentially underestimating the combined pollution effects. Future studies may consider introducing coupled models (e.g., fuzzy–neural networks) or adjusting weights for interaction terms.

(2) Limitations of pollution source estimation. Long-term seasonal sampling and testing were not conducted, so changes in pollution over different periods cannot be reflected. Additionally, pollutant migration and transformation processes, such as sedimentation and degradation within reservoirs, were not considered, which may overestimate the contribution of downstream pollution sources. In the future, seasonal monitoring should be supplemented, a dynamic emission model should be established, and flow data should be obtained in collaboration with hydrological departments to improve the spatio-temporal resolution of pollution sources.

## 4. Conclusions and Recommendations

### 4.1. Conclusions

This study focuses on the water quality of the main stream and tributaries of Wan’an Reservoir. It analyzes the spatial distribution and trends of key water pollutants, employing the entropy weight method and fuzzy membership model to systematically assess the water quality of the reservoir. The following conclusions are drawn:

(1) The water quality in the main stream of Wan’an Reservoir is generally good, as indicated by favorable levels of DO, COD_Mn_, NH_3_-N, and TP. Most sampling points meet the Class II water standard, and some even meet Class I standards. However, the water quality in terms of TN is poor, with 50% of the sampling points being identified as key polluted areas. In some cases, water quality reaches Class V levels.

(2) The water quality of the upper, middle, and lower reaches of the tributaries of Wan’an Reservoir, as indicated by DO, COD_Mn_, NH_3_-N, and TP, generally meets Class II standards, with good water quality overall. However, the TN levels exhibit significant spatial variation, with serious pollution observed throughout the upper, middle, and lower sections. The middle reaches experience the most severe exceedances of the standards.

(3) The water quality of both the main stream and tributaries of Wan’an Reservoir exhibits clear spatial heterogeneity and hierarchical differentiation. Sampling points with Grade I water quality in the main stream are primarily concentrated in the upstream areas, benefiting from better incoming water and less human interference. In contrast, downstream areas experience serious pollution due to the combined effects of domestic sewage, industrial wastewater, and agricultural non-point source pollution. Grade I water quality is also found predominantly in the tributaries’ source areas or regions with minimal human impact. However, downstream tributaries and those flowing through densely populated areas, agricultural zones, and concentrated aquaculture regions are more polluted.

### 4.2. Recommendations

(1) Prioritize remediation of key areas. According to the model results, the tributaries L8, L10, L27, and the downstream area of the main stream WA18 are the most severely polluted and require priority intervention. Therefore, within the L8, L10, and L27 basins, mandatory upgrades of wastewater treatment facilities will be implemented in densely populated aquaculture areas (e.g., Wushu Township near L27) to address pollution at its source. In villages surrounding WA18 (e.g., Baqian Village in Wanan County), centralized sewage treatment networks will be installed to intercept rural decentralized wastewater.

(2) Precise control of pollution sources. According to the results of pollution source analysis, aquaculture is the primary source of TN. Therefore, for aquaculture farms along tributaries such as L27 and L39, feed protein content must be ≤28% to reduce nitrogen emissions. Domestic wastewater is the primary source of COD and TP. Therefore, decentralized wastewater treatment stations should be constructed in the middle reaches of tributaries with TN exceedances (e.g., east of L23). For agricultural non-point sources, it is important to promote the use of controlled-release fertilizers in the WA12-WA15 section (mainstream TN Class V zone) and combine remote sensing monitoring with fertilizer application rates.

(3) Dynamic monitoring optimization. It is necessary to install online water quality sensors in tributaries such as L8, L10, and L27 to closely track changes in TN and COD. Additionally, we recommend dividing the reservoir into three zones—red (L27 and other Class V zones), yellow (WA18 and other Class IV zones), and green (WA1 and other Class I zones)—and implementing differentiated performance evaluations.

## Figures and Tables

**Table 1:** Environmental Quality Standards for Surface Water (GB 3838-2002) vs. customized water quality classification criteria applied in this study.

Water Quality Indicators	Environmental Quality Standards for Surface Water (GB 3838-2002)				
	I	II	III	IV	V
DO (mg/L)	≥7.5	[6.5, 7.5]	[6.0, 6.5]	[3.0, 6.0]	[2.0, 3.0]
TN (mg/L)	≤0.2	(0.2, 0.5]	(0.5, 1.0]	(1.0, 1.5]	(1.5, 2.0]
NH_3_-N (mg/L)	≤0.15	(0.15, 0.5]	(0.5, 1.0]	(1.0, 1.5]	(1.5, 2.0]
TP (mg/L)	≤0.01	(0.01, 0.025]	(0.025, 0.05]	(0.05, 0.1]	(0.1, 0.2]
COD_Mn_ (mg/L)	≤2.0	(2.0, 4.0]	(4.0, 6.0]	(6.0, 10.0]	(10.0, 15.0]
**Water quality indicators**	**Water Quality Grade**				
	**I**	**II**	**III**	**IV**	**V**
DO (mg/L)	≥7.5	(6.5,7.5]	(6.0, 6.5]	(5.5, 6.0]	(4.5, 5.5]
TN (mg/L)	≤0.2	(0.2, 0.5]	(0.5, 1.0]	(1.0, 1.5]	(1.5, 2.0]
NH_3_-N (mg/L)	≤0.15	(0.15, 0.5]	(0.5, 1.0]	(1.0, 1.5]	(1.5, 2.0]
TP (mg/L)	≤0.01	(0.01, 0.025]	(0.025, 0.05]	(0.05, 0.1]	(0.1, 0.2]
COD_Mn_ (mg/L)	≤1.0	(1.0, 2.0]	(2.0, 3.0]	(3.0, 4.0]	(4.0, 5.0]

**Table 2:** Descriptive statistics of water quality indicators (*n* = 83).

Water Quality Indicators	Min	Max	Mean	Std. Dev	Median
DO (mg/L)	4.890	18.360	9.219	2.567	9.250
TN (mg/L)	0.000	18.940	2.277	3.714	1.050
NH_3_-N (mg/L)	0.020	2.740	0.271	0.374	0.180
TP (mg/L)	0.000	0.300	0.031	0.039	0.022
COD_Mn_ (mg/L)	0.000	4.840	1.942	1.236	1.972

**Table 3:** Annual pollutant discharge around Wan’an Reservoir.

Type	Source		Emissions (T)		
		COD_Mn_	NH_3_-N	TN	TP
Industrial source		/	/	/	/
Agricultural source	Livestock and poultry breeding	1146.37	231.85	/	7.25
	Aquaculture	212.65	9.82	30.90	5.69
	Planting industry	/	/	14.01	2.54
Domestic source	Urban life source	1775.75	170.26	233.98	22.30
	Rural life source	546.04	45.73	69.33	5.06
Centralized pollution		1.00	0.10	0.28	0.02
Total		3681.81	457.76	348.51	42.86

## Data Availability

The data that support the findings of this study are available from the corresponding author upon reasonable request.

## Supplementary Materials

The following supporting information can be downloaded at: https://www.mdpi.com/article/10.3390/toxics13090712/s1, Table S1: Normalized fuzzy membership matrix R.
