# Supplementary material for: Water Quality Evaluation and Countermeasures of Pollution in Wan’an Reservoir Using Fuzzy Comprehensive Evaluation Model
Source: Toxics. 2025 Aug 23;13(9):712. doi: 10.3390/toxics13090712 (PMC12473995; doi:10.3390/toxics13090712)
Supplement: Supplementary file 1 [file toxics-13-00712-s001.zip › toxics-3734037-supplementary.pdf]

Table S1 Normalized fuzzy membership matrix R

R=

1	0	0	0	0
0	1	0	0	0
1	0	0	0	0
0	0.41	0.5	0.09	0
0.194444	0.805556	0	0	0
1	0	0	0	0
1	0	0	0	0
0.392857	0.607143	0	0	0
0	0.833333	0.166667	0	0
0	1	0	0	0
0	0.2	0.5	0.3	0
0	1	0	0	0
0	0.531915	0.468085	0	0
0	0.581395	0.418605	0	0
0	0	0	0.904762	0.095238
0	1	0	0	0
0	0.32	0.5	0.18	0
0	0	0	0.513158	0.486842
1	0	0	0	0
0	0	0	0	0
0	0	0	0	0
0	0	0	0	0
1	0	0	0	0
1	0	0	0	0
1	0	0	0	0
0	0	0	0	0
0	0	0	0	0
0	0	0	0	0
0.104839	0.895161	0	0	0
0	0	0	0	0
1	0	0	0	0
0	0	0	0	0
0	0	0	0	0
1	0	0	0	0
0	0	0	0	0
0	0	0	0	0
0	0	0	0	0
1	0	0	0	0
0	1	0	0	0
1	0	0	0	0
0	0.82	0	0.18	0

0.194444	0.805556	0	0	0
1	0	0	0	0
1	0	0	0	0
0.392857	0.607143	0	0	0
0	0.833333	0.166667	0	0
0	1	0	0	0
0	0.2	0.5	0.3	0
0	1	0	0	0
0	0.531915	0.468085	0	0
0	0.581395	0.418605	0	0
0	0	0	0.904762	0.095238
0	1	0	0	0
0	0.64	0	0.36	0
0	0	0	0.513158	0.486842
1	0	0	0	0
0	0	0	0	0
0	0	0	0	0
0	0	0	0	0
1	0	0	0	0
1	0	0	0	0
1	0	0	0	0
0	0	0	0	0
0	0	0	0	0
0	0	0	0	0
0.104839	0.895161	0	0	0
0	0	0	0	0
1	0	0	0	0
0	0	0	0	0
0	0	0	0	0
1	0	0	0	0
0	0	0	0	0
0	0	0	0	0
0	0	0	0	0
1	0	0	0	0
0	0	0	0	0
1	0	0	0	0
0	0	0	0	0
1	0	0	0	0
1	0	0	0	0
